# Hierarchical Sulfide‐Rich Modification Layer on SiO/C Anode for Low‐Temperature Li‐Ion Batteries

**DOI:** 10.1002/advs.202104531

**Published:** 2022-05-07

**Authors:** Xu Liu, Tianyu Zhang, Xixi Shi, Yue Ma, Dawei Song, Hongzhou Zhang, Xizheng Liu, Yonggang Wang, Lianqi Zhang

**Affiliations:** ^1^ Tianjin Key Laboratory for Photoelectric Materials and Devices School of Materials Science and Engineering Tianjin University of Technology Tianjin 300384 China; ^2^ Department of Chemistry and Shanghai Key Laboratory of Molecular Catalysis and Innovative Materials Institute of New Energy iChEM (Collaborative Innovation Center of Chemistry for Energy Materials) Fudan University Shanghai 200433 China

**Keywords:** hierarchical layers, lithium ion batteries, low temperature, SiO/C composite anodes, sulfide‐rich layers

## Abstract

The silicon oxide/graphite (SiO/C) composite anode represents one of the promising candidates for next generation Li‐ion batteries over 400 Wh kg^−1^. However, the rapid capacity decay and potential safety risks at low temperature restrict their widely practical applications. Herein, the fabrication of sulfide‐rich solid electrolyte interface (SEI) layer on surface of SiO/C anode to boost the reversible Li‐storage performance at low temperature is reported. Different from the traditional SEI layer, the present modification layer is composed of inorganic–organic hybrid components with three continuous layers as disclosed by time‐of‐flight secondary ion mass spectrometry (TOF‐SIMS). The result shows that ROSO_2_Li, ROCO_2_Li, and LiF uniformly distribute over different layers. When coupled with LiNi_0.8_Co_0.1_Mn_0.1_O_2_ cathode, the capacity retention achieves 73% at −20 °C. The first principle calculations demonstrate that the gradient adsorption of sulfide‐rich surface layer and traditional intermediate layer can promote the desolvation of Li^+^ at low temperature. Meanwhile, the inner LiF‐rich layer with rapid ionic diffusion capability can inhibit dendrite growth. These results offer new perspective of developing advanced SiO/C anode and low‐temperature Li‐ion batteries.

## Graphical abstract

A sulfide‐rich solid electrolyte is fabricated on the surface of SiO/C composite anode and its reversible Li‐storage performance is improved distinctly. The modified layer is composed by inorganic–organic hybrid components with three continuous layers and is disclosed by time‐of‐flight secondary ion mass spectrometry.

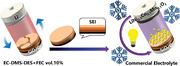

## 1 Introduction

Rechargeable lithium ion batteries (LIBs) occupy the markets of pocket devices, hybrid electric vehicles, and smart grid since their commercialization, and have gained huge economic benefits.^[^ ^1^, ^2^ ^]^ It is generally considered that the increased electrode polarization and decreased ionic diffusion kinetics are associated with the unstable solid electrolyte interface (SEI) layer at lower temperature.^[^ ^3^, ^4^, ^5^, ^6^ ^]^ In addition, the increased Li ions desolvation energy usually leads to formation of Li dendrites on the surface of graphite anode and thus the potential safety hazards.^[^ ^7^, ^8^ ^]^ Silicon‐based materials have 10 times Li ions storage capability than graphite and low reactivity with electrolyte.^[^ ^9^, ^10^ ^]^ It shows a broad prospect as the next generation anode for LIBs. However, the serious expansion of the lithiated Si result in the electrode pulverization and broken of SEI layer,^[^ ^11^ ^]^ which are the main reasons limiting its further application in LIBs. Recently, the fabrication of Si/graphite composite anode has become one of the promising choice which can both improve the energy density and cycle stability of LIBs.^[^ ^12^ ^‐^ ^13^ ^]^

Toward the practical applications of silicon/graphite (Si/C) composite anode, it is still a big challenge to enhance the reversibility of its Li‐storage performance at subzero temperature.^[^ ^11^ ^]^ Great efforts have been devoted to enhancing the low temperature battery performance by regulating the SEI layer at the interface of anode.^[^ ^14^, ^15^, ^16^, ^17^, ^18^, ^19^ ^]^ By fabrication a LiF‐core electrochemical active monolayer on Li metal surface, a stable cycling performance has been achieved due to the changed interface chemical environment.^[^ ^20^ ^]^ Pan et al. modified Li metal anode by a solid electrolyte layer to reduce its reactivity and thus enhancing the durability in assembled cells.^[^ ^21^ ^]^ A high‐modulus LiF‐organic bilayer interphase layer has been constructed through a tetrahydrofuran electrolyte, which can adapt to the volume fluctuation of Si anode during cycling.^[^ ^22^ ^]^ All of the above results remind us the fabrication of suitable SEI layer could distinctly promote the battery performance at low temperature.

We notice that the SEI film formed by carbonate‐based electrolyte is brittle at low temperature and could not bear the large volume expansion of Si‐based composite anode.^[^ ^23^, ^24^, ^25^ ^]^ It is important to build a SEI layer with high mechanical strength and suitable structure which could promote the Li‐desolvation process for Si anode. Some sulfite solvents with high dielectric constant and wide temperature range show excellent compatibility with SiO/C anode, which are usually used as additives of electrolytes to promote the formation of SEI and produce sulfide with low impedance.^[^ ^26^, ^27^, ^28^, ^29^, ^30^, ^31^ ^]^ However, the oxidation of sulfur‐containing solvent at higher potential leads to its continuous consumption and limits its further application in full batteries.^[^ ^32^ ^]^ In addition, the structure and performance‐dependent properties of SEI layer has not been clearly demonstrated which also impede its rational design and optimization.^[^ ^33^ ^]^ Inspired by the above progress, we herein report a new strategy to construct sulfide‐containing SEI layer on the Si‐based anode, and establish a more stable interface between electrolyte and electrode, so as to realize high‐performance SiO/C anode at low temperature. The SiO/C composite anode was assembled into a half cell coupled with Li anode, and the modification layer was formed during the initial discharge process with a sulfite‐contained electrolyte. This modification layer changes the decomposition path and kinetics of Li ions at the interface, thus promoting its desolvation behavior at low temperature. The composition and structure of this layer has been disclosed by time‐of‐flight secondary ion mass spectrometry (TOF‐SIMS), transmission electron microscope (TEM), and X‐ray photoelectron spectroscopy (XPS). Theoretical calculation (DFT) further verified the gradient diffusion process of Li^+^ in organic layer. The SiO/C composite anode with modification layer (M–SiO/C) coupled with a LiNi_0.8_Co_0.1_Mn_0.1_O_2_ cathode demonstrate a capacity retention of 73% at −20 °C, and after 200 cycles, the capacity retention rate still remains 95.3% of the 5th cycle. This work sheds light on the deeper understanding of the interface chemistry and provides rational scientific principles in designing the electrode/electrolyte interface for long‐term cycling rechargeable batteries.

## 2 Results and Discussion

**Figure 1:**
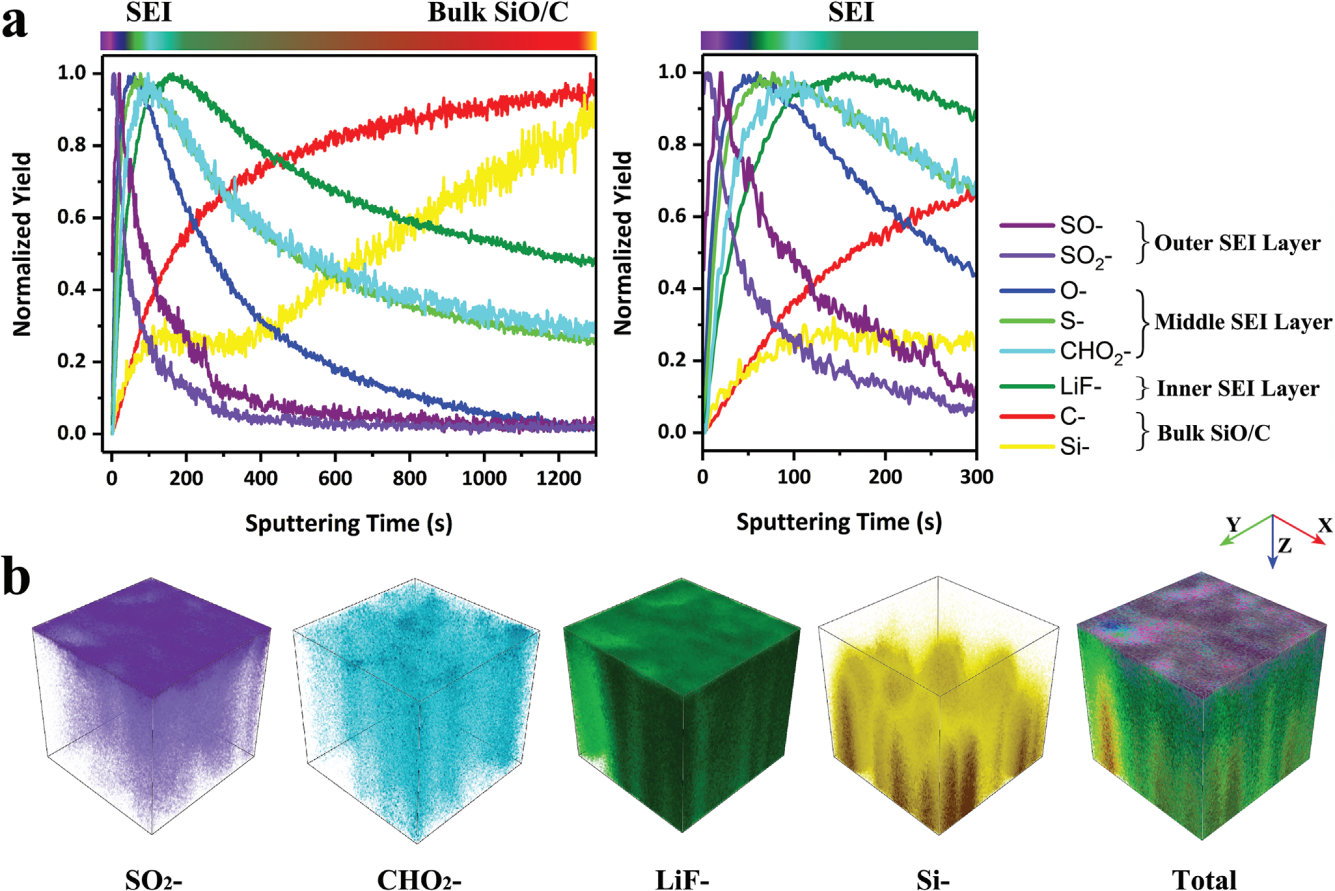
TOF‐SIMS analysis of the modified layer. a) Normalized (to their maximum) depth profiles of various secondary ions of interest contained in the sputtered volume at the modified layer. b) Individual spatial distributions of the selected secondary ions (SO_2_—, CHO_2_—, LiF—, and Si—) and 3D, multicolor overlay of representative secondary ions composing the different layers of the interface.

The surface modification layer for SiO/C anode was electrochemically formed within a half cell. The SiO/C composite anode coupled with Li metal were assembled into half cells. The cells were subjected to cycle at 0.1C in EC/DMS/DES+FEC vol10% electrolyte. After certain cycles, the cells were disassembled and the SiO/C composite electrodes at lithiated states (named as M—SiO/C) were collected for further characterizations or anodes in full cells. Similarly, the SiO/C without surface modification but after a certain cycle in commercial electrolyte (LiPF_6_‐EC/DMC) (named as N—SiO/C) is also used for comparison. TOF–SIMS was adopted to clearly disclose the composition and distribution of modification layer in M—SiO/C.^[^ ^34^ ^]^ The normalized depth profiles in Figure S1a (Supporting Information); and **Figure** **1** shows the composition of surface layer after processing with two different electrolytes, respectively. In Figure S1a (Supporting Information), the SEI film in normal electrolyte composed of organic–inorganic double layer which has been revealed previously. At the surface, the contents of CH_2_, CO_2_—, and O— are observed and their intensities increase first, indicating that the organic layer formed at the interface and mainly composed of alkoxy carbonate (ROCO_2_—). With the deepening of sputtering, the content of LiF— increases continuously, while the CO_2_— curve also drops sharply and then rises. This is consistent with the trend of Li— curve, indicating the emergence of inorganic components such as Li_2_CO_3_ and LiF. The rising rate of O— curve slows down, which should be associated with the formation of Li_2_O. Figure 1a displays the SEI contents of M—SiO/C. Three‐layer characteristics can be clearly observed. The outer layer displays high contents of SO— and SO_2_— which means the DMS and DES has been reduced to ROSO_2_Li. This sulfide‐rich layer could promote the stability of SEI by inhibiting the successive decomposition of electrolyte at the surface of anode. With the deepening of the profile, the contents of O—, S—, and CHO_2_— increase, among which O— and CHO_2_— provide evidence for the presence of alkoxy carbonate (ROCO_2_Li) in this SEI. The variation of S— and CHO_2_— contents are almost identical, which proves the formation of alkylthio compounds (RCH(OSO_2_Li)CH_2_OCO_2_Li) in the middle layer of SEI. As the main organic component of traditional SEI layer, ROCO_2_Li has good ionic conductivity but poor mechanical strength. The introduction of —OSO_2_Li branch will reduce the crystallinity and increase the elastic modulus. After etching for 100–300 s, it can be seen that CHO_2_— content decreases while LiF— content increases and remains at a high level. This result indicates the LiF‐rich inner layer is close to the anode surface. LiF which is ionic conducive could regulate the uniform deposition of Li^+^ at low temperature. 3D views of SEI layer directly visualize the homogeneous coverage of trimolecular layer feature (Figure 1b). Compared with the traditional SEI layer in Figure S1b (Supporting Information), the products of each layer in the modified layer are more uniform, whereby reversible redox reactions at low temperature environment can be expected.^[^ ^35^ ^]^

**Figure 2:**
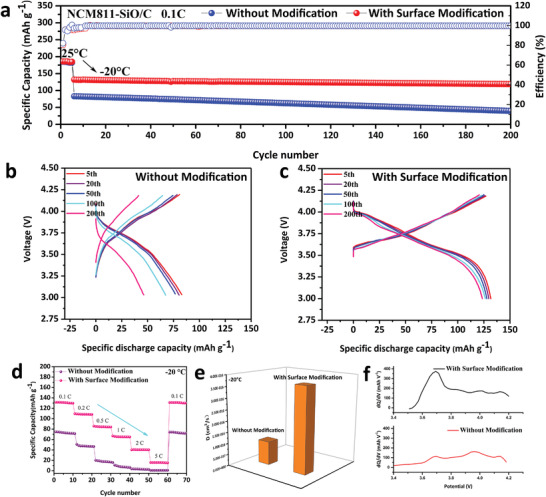
a) Long cycle performance of two kinds of batteries at −20 °C. b,c) Charge–discharge curves of two kinds of batteries at −20 °C. d) Rate performance of two types of batteries at −20 °C. e) Solid phase diffusion coefficient of Li^+^ in different anodes. f) D*Q*/d*V* curves of two types of batteries at −20 °C.

The Li storage performance of M—SiO/C composite anode coupled with LiNi_0.8_Co_0.1_Mn_0.1_O_2_ cathode has been carefully studied both at room temperature and −20 °C. The full cells with LiNi_0.8_Co_0.1_Mn_0.1_O_2_ and N—SiO/C were assembled for comparison. Figure S2a (Supporting Information) shows the long cycle performance at room temperature. It can be clearly observed that the specific discharge capacities of the two batteries are about 185 mAh g^−1^ in the initial cycle. There is an obvious capacity decay of the battery with fresh cathode and N—SiO/C anode during cycling. It is only 150 mAh g^−1^ after 50 cycles, and gradually decreases to about 100 mAh g^−1^ after 200 cycles. The fast capacity decay at the early stage of cycle may be associated with the gradual stacking of SEI layers.^[^ ^36^ ^]^ As the cycle continues, the side‐reaction between electrolyte and electrodes tends to be stable, thus delaying the capacity decay rate.^[^ ^37^, ^38^, ^39^, ^40^ ^]^ The battery with M—SiO/C anode shows an improved cycle stability with discharge specific capacity of 175 mAh g^−1^ after 50 cycles, about 168 mAh g^−1^ after 100 cycles, and 155 mAh g^−1^ after 200 cycles, respectively. The modified SEI layer can inhibit the passivation reaction between electrolyte and SiO/C anode at the early stage of cycle, and promote the diffusion kinetics of lithium ions, thus the battery achieves a long cycle life.^[^ ^41^, ^42^ ^]^ **Figure** **2**a shows the long cycle performance at −20 °C (0.1 C). The discharge capacity of the battery with N—SiO/C anode rapidly reduces to 80 mAh g^−1^ after the first several cycles, and the capacity retention rate is 43% of that at room temperature. The discharge capacity decays to 68 mAh g^−1^ after 100 cycles and then to 48 mAh g^−1^ after 200 cycles with capacity retention rate of only 26%. By comparison, the battery with M—SiO/C anode shows a better low‐temperature performance. The Coulombic efficiency of the battery is 82% in the initial cycle, and quickly increases to 99.9% in the following cycles. It is proved that the modified SEI layer has good compatibility with the commercial electrolyte. The discharge specific capacity is 135 mAh g^−1^ for the initial cycle at −20 °C, and the capacity retention rate is 73% of that at room temperature. After 100 and 200 cycles, the capacity remains 129 mAh g^−1^ (69.7%) and 123 mAh g^−1^ (66.5%), respectively.

Figure S2b,c (Supporting Information) shows the selected cycles of voltage profiles of the two batteries at room temperature. Initially, both of them display similar discharge plateau at about 3.65 V. After 200 cycles, the plateau degrades to about 3.5 V for the battery with N—SiO/C anode, which is lower than that of 3.6 V with M—SiO/C anode. This is due to the successive electrolyte decomposition, formation of by‐products, and uneven SEI layer on the surface of the SiO/C anode, which result in serious electrode polarization and high impedance. This also destroys the diffusion kinetics of Li^+^.^[^ ^6^ ^], [^ ^22^ ^], [^ ^43^ ^]–[^ ^45^ ^]^ The M—SiO/C anode exhibits excellent compatibility with commercial electrolyte and demonstrates low ionic diffusion barrier. The charging and discharging curves at low temperature are shown in Figure 2b,c. As the temperature down to −20 °C, the discharge plateau of the battery with N—SiO/C anode decay to 3.5 V, and gradually decrease to 3.25 V which distinct the successive electrode polarization upon cycling. Meanwhile, the discharge plateau for the batteries with M—SiO/C anode is about 3.6 V initially and remains 3.55 V even after 200 cycles. The results indicate that the by‐products accumulation on the surface of N—SiO/C anode during cycling, which affects the diffusion kinetics of Li^+^ at low temperature and leads to the increase of electrode polarization. The smooth diffusion of Li^+^ ions within the modified layer of M—SiO/C anode results in a lower charge transfer resistance both at room temperature and lower temperature.^[^ ^46^, ^47^ ^]^ This may be associated with that the sulfur‐rich modification layer could facilitate the desolvation process especially at lower temperature and thus suppressing the deterioration of electrode polarization.^[^ ^14^ ^], [^ ^48^ ^]–[^ ^50^ ^]^

Figure 2d shows the rate performance at −20 °C. For the batteries with N—SiO/C anode, the discharge capacity is about 75 mAh g^−1^ at 0.1 C, and it quickly decreases to 48 and 20 mAh g^−1^ at 0.2 C and 0.5 C, respectively. Meanwhile, the batteries with M—SiO/C shows distinctly improved performance. The capacity is about 135 mAh g^−1^ at 0.1 C, and remains 110 mAh g^−1^ at 0.2 C. Even at 1 C and 2 C, the discharge specific capacity remains at 60 and 40 mAh g^−1^. The voltage profiles are shown in Figure S3a,b (Supporting Information). The discharge plateau of the battery with N‐SiO/C anode is about 3.5 V at 0.1 C, and drops to 3.3 V at 0.2 C. When the rate exceeds 0.5 C, there is no obvious charge–discharge plateaus due to the large electrode polarization. The discharge plateaus for the battery with M—SiO/C are 3.6 (0.1 C), 3.5 (0.2 C), and 3.25 V (0.5 C), respectively. Comparing the discharge curves of the two batteries, it is found that the end slope of the discharge curve of the battery with M—SiO/C is larger than those of the batteries with N—SiO/C anode. These results suggest that the charge transfer in electrolyte at low temperature may not be the main factor affecting the electrode polarization, while modification of Li desolvation at the surface of anode is critical for the battery performance at low temperature.^[^ ^8^ ^], [^ ^51^ ^]–[^ ^52^ ^]^ In order to further verify the low temperature cycle performance, M—SiO/C anode and N—SiO/C anode coupled with lithium metal in commercial electrolyte for long cycle were tested. As shown in Figure S4a (Supporting Information), after the 10th cycle, the capacity of the N—SiO/C anode rapidly decayed to 35 mAh g^−1^ and remains at a low level, while the discharge capacity of the M—SiO/C anode exceeds 375 mAh g^−1^, and remains about 275 mAh g^−1^ after 100 cycles. The corresponding charge–discharge curves are shown in Figure S4b,c (Supporting Information), and the N—SiO/C anode loses its stable charge–discharge platform at the initial stage of cycle. However, the M—SiO/C anode shows stable charge–discharge platforms at 0.25 and 0.45 V, 0.1 and 0.2 V, respectively, and there is no obvious increasing of polarizations after 100 cycles. It is proved that M—SiO/C anode has highly reversible lithiation/delithiation behavior, and the modified layer still has excellent stability at low potential.

In order to explore the influence of SEI layer on the dynamic behavior of Li^+^ at low temperature, we carried out EIS measurement of two batteries after 50 cycles at −20 °C. As shown in Figure S5a (Supporting Information), the high frequency region corresponds to the resistance of Li^+^ passing through SEI film, in which the resistance of SEI film on N—SiO/C anode directly generated in commercial electrolyte is about 50 Ω. It is only 25 Ω for the anode with a premodified layer. The *R*_CT_ corresponds to the charge transfer resistance from SEI layer to electrode, which is 175 and 63 Ω, respectively. In order to further explore the diffusion kinetics of Li^+^, we calculated the diffusion coefficient of Li^+^ by Equation (1)

(1) $$D_{Li^{+}}=\;0.5{\left(\frac{\mathrm{RT}}{n^{2}F^{2}\mathrm{AC}A_{w}}\right)}^{2}$$

In this equation, “R” is the gas constant, “T” is kelvin temperature, “A” is the surface area of the electrode, “n” represents the number of the electrons per molecule attending the electronic transfer reaction, “F” is the Faraday constant, “C” represents the bulk concentration of Li^+^, and “A_w_” is the Warburg coefficient. The diffusion coefficient within different anodes is shown in Figure 2e, which demonstrates an order of M—SiO/C (3.75 × 10^−16^ cm^2^ s^−1^) > N—SiO/C (1.04 × 10^−16^ cm^2^ s^−1^). The higher coefficient with M—SiO/C anode demonstrates a smooth charge‐transfer process across the electrolyte/electrode interface. This mainly benefits from reduced interface impedance and energy barrier. On the other hand, it also shows that the modified layer improves the desolvation behavior and ion migration rate of Li^+^ during the intercalation process. Figure 2f is the corresponding d*Q*/d*V* curve at −20 °C. Sharp peaks can be observed and the improved lithiation/delithiation kinetics is mainly due to the sulfur‐rich modification layer at low temperature.

**Figure 3:**
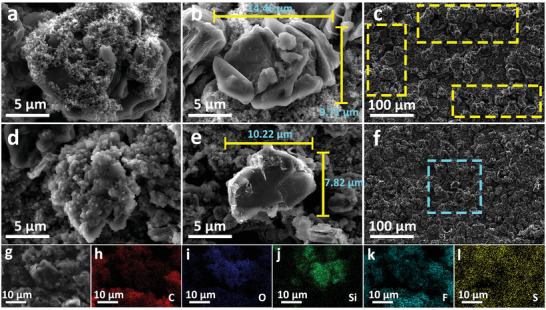
SEM image of SiO/C anode a–c) without and d–f) with modified layer after 50 cycles at −20 °C. g–l) EDS image of SiO/C with modified layer after cycling at −20 °C.

The evolution of surface morphology of cycled electrodes has been examined and the SEM images of the two anodes after 50 cycles at −20 °C are shown in Figure 3. Without premodified layer, there is an incomplete and loose SEI film on the surface of SiO/C (**Figure** **3**a). Large number of holes on the surface of the film can be observed. Figure 3b shows the morphology of pure SiO particles after cycling. Compared with the fresh SiO particles (Figure S6a, Supporting Information), the particles become significantly larger and some of them break into smaller particles. As shown in Figure 3c, there are large cracks appeared, while a clean surface for the fresh anode in Figure S6b (Supporting Information). This should be associated with the serious expansion of SiO accompanied with the lithiation. Large‐volume particles gradually break away from the constraints of graphite lattice and bulge on the surface, and exhibit a high tendency to peel off the electrode. The SEM image of cycled M—SiO/C anode at −20 °C is shown in Figure 3d. The complete and compact morphology of the modified layer is clearly seen from the surface. Comparing with the fresh modified electrode (Figure S7a,b, Supporting Information), there is no obvious difference except some raised particles, which indicates the modified layer has excellent stability and compatibility with commercial electrolyte. For single SiO particles (Figure 3e), there is only slight corrosion on the particle surface, without obvious expansion and smash. From Figure S8a,b (Supporting Information), it can be seen that under low magnification, there are several SiO particles with similar morphology in a certain range, which proves that the volume change of SiO particles in the two systems conforms to the typical characteristics described. There are no obvious cracks observed at lower magnification in Figure 3f. These results clearly show that the modified layer has good ductility and mechanical strength even at low temperature. Moreover, it protects the graphite layer from falling off. We also collected elemental mapping of M—SiO/C anode by energy dispersive spectrometer (EDS) (Figure 3g–l). The uniformly distributed F and S indicate that the modified layer is rich in compounds containing these two elements.

**Figure 4:**
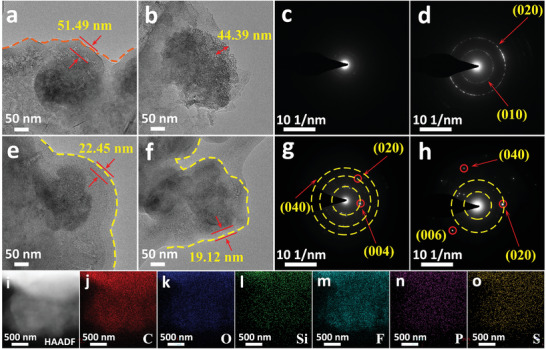
TEM images of a) charged and b) discharged SiO/C anode of the brand‐new full battery after cycling at −20 °C. SAED image of corresponding c) charged and d) discharged SiO/C anode in brand‐new full battery. TEM images of e) charged and f) discharged SiO/C anode with modified layer after cycling at −20 °C. SAED image of corresponding g) charged and h) discharged SiO/C anode with modified layer. i–o) The EDS images of the SiO/C anode with modified layer after cycling at −20 °C.

TEM further studied the detailed morphology and structural information of SEI film at low temperature. The fresh SiO/C composite electrode shows a clear and clean surface, as shown in Figure S9a,b (Supporting Information). The morphology of particles on the lithiated N—SiO/C anode at −20 °C is shown in **Figure** **4**a, and the particle surface shows uneven and thick SEI layer with a thickness about 51.49 nm. It means that the electrolyte is constantly decomposed at the electrode interface, resulting in an increase in the SEI layer thickness. Broken and irregular areas are frequently distributed on the surface, which is due to the lack of toughness of SEI layer, leading to breakage and shedding. However, upon repeated cycling, the SEI layer on the particle surface is seriously damaged (Figure 4b). Uneven deintercalation of Li^+^ makes the SEI film unable to bear large stress, resulting in large‐scale shedding and decomposition. This process leads to a certain degree of decomposition, and irregular SEI layer with large number of pores. Figure 4c,d is the selected area electron diffraction (SAED) images of SiO/C anode at the stage of lithiation and delithiation, respectively. The polycrystalline rings can be clearly seen after delithiation. The interplanar crystal spacing of (020) is about 0.17 nm, which is about 0.06 nm wider than that of the standard (020) crystal plane (0.11 nm), indicating a serious solvent‐ion cluster cointercalation into graphite layers, which leads to the unrecoverable lattice distortion. Figure S9c,d (Supporting Information) shows the modified layer of about 20 nm thickness on the M—SiO/C surface before cycling. Figure 4e displays the TEM image of lithiated M—SiO/C anode at −20 °C. There is a uniform and complete SEI layer with a thickness of about 22.45 nm on the particle surface, which is only half that of the SEI layer on the surface of N—SiO/C anode. There is no obvious change in the thickness and morphology of the SEI film after lithium removal (Figure 4f). The results show that the modified layer has excellent stability, and Li^+^ has high deposition uniformity at low temperature. The amorphous morphology of the electrode surface indicates that the modified layer has good compatibility with the commercial electrolyte. The corresponding SAED image in the delithiated state (Figure 4g) shows a clear polycrystalline ring, and the distance between the (020) crystal planes is about 0.12 nm, which is only about 0.01 nm wider than the standard (020) crystal plane. The corresponding SAED patterns are shown in Figure 4h. Clear diffraction spots and polycrystalline rings reflect that the crystallinity of composite anode keeps well. The sulfur‐rich modified layer promotes the desolvation process of the Li ions both at room temperature and lower temperature during repeated Li ions intercalation/deintercalation, also alleviates the lattice distortion caused by dead Li accumulation in normal conditions. Figure 4i–o is EDS mappings of C, O, Si, F, P, and S corresponding to the M—SiO/C anode particles after cycling. Comparing with the unmodified anode (Figure S10a–f, Supporting Information).), we find that the elements on the surface of modified SiO/C anode particles distribute more evenly, and the signal of F element is stronger, which is associated with the embodiment of LiF in SEI film. Moreover, the uniform distribution of S elements on the particle surface proves that there is abundant —OSO_2_— in the organic layer.

**Figure 5:**
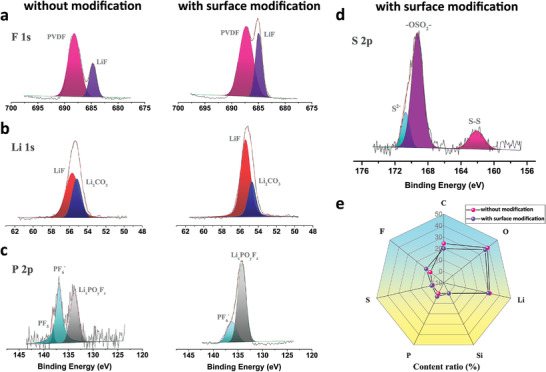
a–d) XPS profiles of F 1s, Li 1s, P 2p, S 2p spectra and e) atomic surface concentration on the SiO/C anodes of two kinds of batteries after 50 cycles at −20 °C.

XPS was used to explore the composition and element valence state of the SEI layers on the surface of anode after cycles at −20 °C. **Figure** **5**a shows the F 1s spectrum. The peak at 688.0 eV corresponds to the C—F bond from poly(vinylidene fluoride) (PVDF) adhesive, and LiF appears at 685.5 eV. Combined with the intensity of Li 1s spectrum in Figure 5b, it can be clearly seen that the LiF (56.5 eV) signal on the surface of the modified SiO/C anode is stronger, which is consistent with F spectrum. The large amount of LiF could promote the uniform transportation of Li to access SiO/C anode, while the present of Li_2_CO_3_ (55.5 eV) could increase the electronic insulation.^[^ ^53^ ^]^ Figure 5c shows the spectrum of P 2p which originates from Li*_x_* PO*_y_* F*_z_* (134.6 eV) with high content. According to Equations (1) and (2), it can be determined as LiPO_2_F_2_, the decomposition product of LiPF_6_. LiPO_2_F_2_ is an excellent film‐forming additive for both cathode and anode.^[^ ^54^, ^55^, ^56^ ^]^ Furthermore, LiPO_2_F_2_ deposited on the electrode surface will be further decomposed into Li_3_PO_4_ and LiF (Equation (3)), thus effectively reducing the interfacial side reactions of the electrode.^[^ ^57^, ^58^ ^]^ More PF_5_ (137.7 eV) that can be observed on the surface of SiO/C anode without modification layer, is another product after LiPF_6_ decomposition (Equation (4)). However, PF_5_ could react with H_2_O and decompose into HF (Equation (5)), thus further destroying the SEI layer as well as anode. This indicates that sulfide in the modified layer leads the directional reaction with LiPF_6_ and inhibits the formation of harmful by‐products. S 2p spectra collected from the modified SiO/C anode in Figure 5d consist of S^2−^ at 170.7 eV, —OSO_2_— at 169.3 eV and S—S bond at 164 eV. The S^2−^ is associated with the Li_2_S product. The —OSO_2_— and S—S bond represent the organic components in the modified layer, which is consistent with the TOF–SIMS data. It is proved that the modified layer does not decompose on a large scale during cycles and has excellent compatibility with commercial electrolytes. Further quantitative evidence supporting these conclusions could also be found in Figure 5e

(2) $$\mathrm{LiP}F_{6}+H_{2}O\to \mathrm{LiF}↓+2\mathrm{HF}+\mathrm{PO}F_{3}\;$$

(3) $$\mathrm{PO}F_{3}+ne^{-}+nLi^{+}\to \mathrm{LiF}↓+Li_{x}PO_{y}F_{z}$$

(4) $$PO_{2}{F_{2}}^{-}+ne^{-}+nLi^{+}\to Li_{3}PO_{4}↓+\mathrm{LiF}↓$$

(5) $$\mathrm{LiP}F_{6}↔\mathrm{LiF}↓+PF_{5}$$

(6) $$PF_{5}+H_{2}O\to \mathrm{PO}F_{3}+2\mathrm{HF}$$

**Figure 6:**
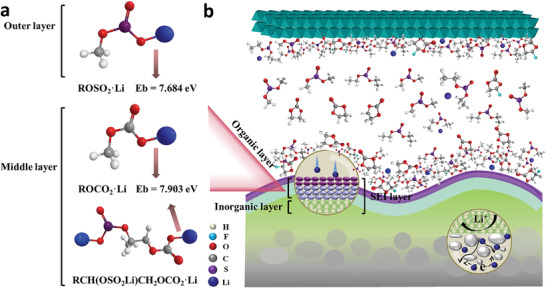
a) Binding energy for Li^+^ with organic components in modified layer by DFT. b) Simulation model of molecular dynamics in battery.

In order to further illustrate the advantage of modified layer over the traditional SEI layer, we conducted the density functional theory (DFT) calculations to disclose the interactions between the surface layer and Li^+^, as shown in **Figure** **6**a. According to the characterization results, the most possible structure of organic components in modified layer includes two layers. The outer layer is mainly composed of CH_3_OSO_2_Li, while the middle layer is CH_3_OCO_2_Li and CH_3_CH(OSO_2_Li)CH_2_OCO_2_Li. To simplify the theoretical calculations, the binding energy of Li^+^ with CH_3_OSO_2_— and CH_3_OCO_2_— has been calculated. The binding energy *E*_b_ of Li^+^ interacting with the dual‐layered interface can be defined as the energy difference between the total energies of Li^+^ *E*_Li_ and the organics of the dual‐layered film *E*_organics_,^[^ ^59^ ^]^ that is, *E*_b_ = *E*_Li_ + *E*_organics_ − *E*_Li+organics_. The *E*_b_ of CH_3_OSO_2_—Li is 7.684 eV, which is lower than that of CH_3_OCO_2_—Li (7.903 eV). Lower binding energy means a higher ionic conductivity. In addition, the binding energy between Li^+^ and TFSI— is −6.04 eV (due to the electrostatic interaction). Actually, due to solvation effect, the combination of Li^+^ and TFSI— may not be the main state of Li^+^ in electrolyte.^[^ ^60^ ^]^ And the binding energy calculated above is far greater than that between Li^+^ and solvent components, indicating that Li^+^ can be desolvated from the clusters within the organic layer. The binding energy of Li^+^ with the outer layer is smaller than that of Li^+^ with middle layer, which means that Li^+^ diffuses gradiently in the first two layers. Meanwhile, the high content of Li_3_PO_4_ in the inner inorganic layer is a fast lithium ion conductor, which also provides high ionic conductivity of about 10^−6^ S m^−1^. It is much higher than that of LiF (= 3 × 10^−9^ S m^−1^) and Li_2_CO_3_ (= 10^−8^ S m^−1^),^[^ ^61^, ^62^ ^]^ two typical inorganic components in traditional SEI. As a result, the modified layer exhibits excellent Li‐ion conductivity as well as low internal resistance. In order to further clarify SEI chemistry at low temperature, molecular dynamics simulation was carried out as shown in Figure 6b. The delithiation behavior of solvated lithium ion clusters on the surface of the modified layer can be seen. When a higher potential is applied, Li^+^ enrichment at the electrode/electrolyte interface increases. It also means that the chemical environment of the interface plays an indispensable role in the desolvation of lithium ions at low temperature.^[^ ^63^ ^]^

## 3 Conclusions

In summary, we have developed a sulfur‐rich SEI layer for SiO/C composite anode which boosted Li‐storage performances of low‐temperature batteries. This SEI layer was obtained through precycling of the SiO/C anode in 1 m LiTFSI‐EC/DMS/DES+FEC 10 vol% electrolyte system. This layer possesses high interface energy and mechanical strength to accommodate the large volume change of the SiO/C composite anode. The present modification layer is composed of inorganic–organic hybrid components with three continuous layers as disclosed by TOF–SIMS. The result shows that ROSO_2_Li, ROCO_2_Li, and LiF uniformly distribute over each layer. When coupled with cathode of LiNi_0.8_Co_0.1_Mn_0.1_O_2_, the capacity retention achieves 73% at −20 °C. After 200 cycles, the capacity retention is still 95.3% of the 5th cycle. The sulfide‐rich organic layer effectively prevents the corrosion of active substances by decomposition products of commercial electrolyte and guides the formation of LiPO_2_F_2_, while inorganic layer rich in LiF promotes the uniform deposition of Li^+^ at low temperature. The uniform Li ions deposition and gradient diffusion behaviors through the organic layers have been further verified by DFT calculations. We believe that our strategy of fabrication surface sulfur‐rich SEI layer make great contribution in boosting the low temperature Li‐storage, and shows good prospects for all‐climate batteries.

## Conflict of Interest

The authors declare no conflict of interest.

## Supporting information

Supporting information

## Data Availability

Research data are not shared.
