# Supplementary material for: Hierarchical Sulfide‐Rich Modification Layer on SiO/C Anode for Low‐Temperature Li‐Ion Batteries
Source: Adv Sci (Weinh). 2022 May 7;9(20):2104531. doi: 10.1002/advs.202104531 (PMC9284185; doi:10.1002/advs.202104531)
Supplement: Supplementary file 1 — Supporting information [file ADVS-9-2104531-s001.pdf]

ADVANCED SCIENCE
Open Access

Supporting Information

for *Adv. Sci.*, DOI 10.1002/advs.202104531

Hierarchical Sulfide-Rich Modification Layer on SiO/C Anode for Low-Temperature Li-Ion Batteries

Xu Liu, Tianyu Zhang, Xixi Shi, Yue Ma, Dawei Song, Hongzhou Zhang, Xizheng Liu*, Yonggang Wang and Lianqi Zhang*

Support Information

Hierarchical sulfide-rich modification layer on SiO/C anode for low-temperature Li-ion batteries

Xu Liu[1], Tianyu Zhang[1], Xixi Shi[1], Yue Ma[1], Dawei Song[1], Hongzhou Zhang[1], Xizheng Liu[1]*, Yonggang Wang[2] and Lianqi Zhang[1]*

1 Tianjin Key Laboratory for Photoelectric Materials and Devices, School of Materials Science and Engineering, Tianjin University of Technology, Tianjin 300384, China

2 Department of Chemistry and Shanghai Key Laboratory of Molecular Catalysis and Innovative Materials, Institute of New Energy, iChEM (Collaborative Innovation Center of Chemistry for Energy Materials) Fudan University, 200433 Shanghai, China.

AUTHOR INFORMATION

*Corresponding Author: shixixi_tjut@163.com

xzliu@tjut.edu.cn

Experimental Section

Materials. Ethylene carbonate (EC) (purity: >99.0% GC), fluoroethylene carbonate (FEC) (purity: >99.0%), dimethyl sulfite (DMS) (purity: 99%), diethyl Sulfite (DES) (purity: 98.0%) and Bis(trifluoromethane)sulfonimide lithium salt (LiTFSI) (purity: 99%) were commonly purchased through Aladdin company. $LiNi_{0.8}Co_{0.1}Mn_{0.1}O_2$, graphite and silicon oxide were provided from Tianjin Jiewei Battery Joint-Stock Co., Ltd. Commercial electrolyte ($LiPF_6$-EC/DMC) was provided from Tianjin Jinniu power supply material co., ltd.

Electrode Preparation. For fabrication of anode electrodes (SiO/C), graphite and SiO were mechanically mixed at a weight ratio of 9:1, and then mixed with acetylene black and PVDF (8:1:1 in weight) in N-methyl-2-pyrrolidone (NMP). The nominal specific capacity of SiO/C composite anode is 464.8 mAh g^{-1} (the nominal specific capacity of graphite is 372 mAh g^{-1}, the nominal specific capacity of SiO is 1300 mAh g^{-1}, the ratio is 9:1). The obtained slurry was coated onto Cu foil and dried at 60 °C for 24 h, followed by a roll-pressing. For fabrication of cathode electrodes, the as-prepared active materials ($LiNi_{0.8}Co_{0.1}Mn_{0.1}O_2$) were mixed with acetylene black and PVDF (8:1:1 in weight) in N-methyl-2-pyrrolidone (NMP). The nominal specific capacity of $LiNi_{0.8}Co_{0.1}Mn_{0.1}O_2$ is 185 mAh g^{-1}. The obtained slurry was coated onto Al foil and dried at 60 °C for 24 h, followed by a roll-pressing. Before use, the

electrodes were dried again in a 120 °C vacuum drying oven for 12 h and punched in 16 mm diameter disks. The loading level of active materials in the electrode is about 2.65 mg cm^{-2}.

Construction of Modified Layer. The electrolyte with lithium salt concentration of 1 mol L^{-1} was prepared by dissolving LiTFSI in the solvent of EC: DMS: DES: FEC=3:3:3:1. The half cells assembled with blank SiO/C as working electrode and metallic lithium (16 mm) as counter electrode were charged and discharged in the electrolyte of 1M LiTFSI-EC/DMS/DES+FEC 10 vol% system for 5 cycles at room temperature and 0.1 C rate. Then the SiO/C electrode with the modified layer was removed.

Cycle Scheme. The first step is to assemble the SiO-Li half-cell, and then charge and discharge in the EC-DMS-DES+FEC 10 vol% electrolyte at room temperature (25 °C) at a current density of 0.1 C. In order to ensure that the formed SEI layer is sufficiently stable, the modified anode (M-SiO) was taken out after the battery was cycled 5 times at room temperature. The SOC state of the half-cell is 100%. The removed M-SiO and fresh $LiNi_{0.8}Co_{0.1}Mn_{0.1}O_2$ cathode are assembled into a full battery in a commercial electrolyte (EC-DMC). In order to avoid additional side reactions caused by the low temperature environment, the reconstituted full battery is also charged and discharged for 5 cycles at a current density of 0.1 C at room temperature and then directly cooled to -20 °C without standing still to continue the

cycle.

Electrochemical Characterization. The electrodes were electrochemically characterized using 2032 type of coin cells. The full cell was assembled in commercial electrolyte with SiO/C composite electrode as anode and $LiNi_{0.8}Co_{0.1}Mn_{0.1}O_2$ as cathode. Celgard film was employed as the separator. 1 C is considered as 185 mA g^{-1} of active material. The coin cells were assembled in an argon-filled glove box (MBraun, H_2O <0.1 ppm, O_2 <0.1 ppm). The galvanostatic discharge–charge measurements were performed in the potential range of 3–4.2 V on a Land battery test system (Land CT2001A) at room temperature and at -20 °C (High and low temperature incubator, TC-S8521,TERCHY,China). Impedance data were collected by using an electrochemical workstation (CHI 760E).

Characterizations. After circulation, the coin cells were disassembled and cleaned with ethylene glycol dimethyl ether (DME) to analyze the morphology and surface composition changes of the SiO-C electrode. The surface element information before and after the cycling was collected by time-of-flight secondary ion mass spectrometer (TOF-SIMS,TOF.SIMS5-100) and X-ray photoelectron spectrometer (XPS, Escalab 250Xi, ThermoFisher Scientific). The morphology and structure of the electrode in the discharge state were mapped by a high-resolution scanning electron microscope (HRSEM, Verios 460L, FEI) with FEG, a high-resolution transmission electron microscope (HRTEM, Talos F200 X, FEI) with FEG and an energy dispersive x-ray

spectroscopy (EDS).

Computational Details. Molecular Dynamics (MD) simulations of the three lithium ion electrolyte systems were performed using Gromacs program suite[1] with the all-atom optimized potentials for liquid simulations (OPLS-AA) force field. The OPLS-2009IL force field parameters of PF_6^- and $TFSI^-$ anions were obtained directly from a literature[2] and a charge scaling of 0.8 was adopted to mimic polarization and charge transfer effects. The force field parameters of other neutral organic molecules were generated using LigParGen web server[3]. The 1.2-scaling CM5 charges were based on DFT calculation by Gaussian program [Gaussian, Inc., Wallingford CT, 2016.] and Hirshfeld population analysis by Multiwfn[4].

The initial simulation boxes of dimensions 50×50×50 $Å^3$ packed with electrolyte components were constructed using packmol program[5]. There structures were first relaxed by energy minimizing calculations, and then undergo an annealing from 0 to 298.15 K with the time step of 1ps during 1ns to reach the equilibrium state. Velocity-rescale thermostat[6] with a relaxation constant of 1 ps was used to control the temperature at 298.15 K. Berendsen's barostat with a isothermal compressibility constant of 4.5×10^{-5} was used to control the pressure at 1.01325×10^{5} Pa. Periodic boundary conditions were applied in all directions. Particle-mesh Ewald (PME) method with a cut-off distance of 10 Å was applied to treat the electrostatic interactions and the van der Waals forces.

Upon quasi-equilibrium of the system, a MD simulation for a total simulation

time of 50 ns were performed at constant NPT ensemble, and the trajectory was saved every 10 ps. we further counted the Li-Sol clusters using Visual Molecular Dynamic program (VMD) [Humphrey, W., Dalke, A. and Schulten, K., "VMD - Visual Molecular Dynamics", J. Molec. Graphics, 1996, vol. 14, pp. 33-38.] and Tcl scripts written by ourselves.

The geometries of MSO- and MCO- were optimized under the framework of density of functional theory (DFT) with PBE0 functional[7] and def2SVP basis set[8]. All these DFT calculations were performed using Gaussian 09 program suite. The absorb energy of Li^+ cation with SEI components, such as MSO- and MCO- were calculated according to the formula below:

$$E\_abs = E\ (Complex) - E\ (Li^+) - E\ (SEI)$$

Where E (Complex) is the total energy of the complex when Li^+ adsorbed on the SEI.

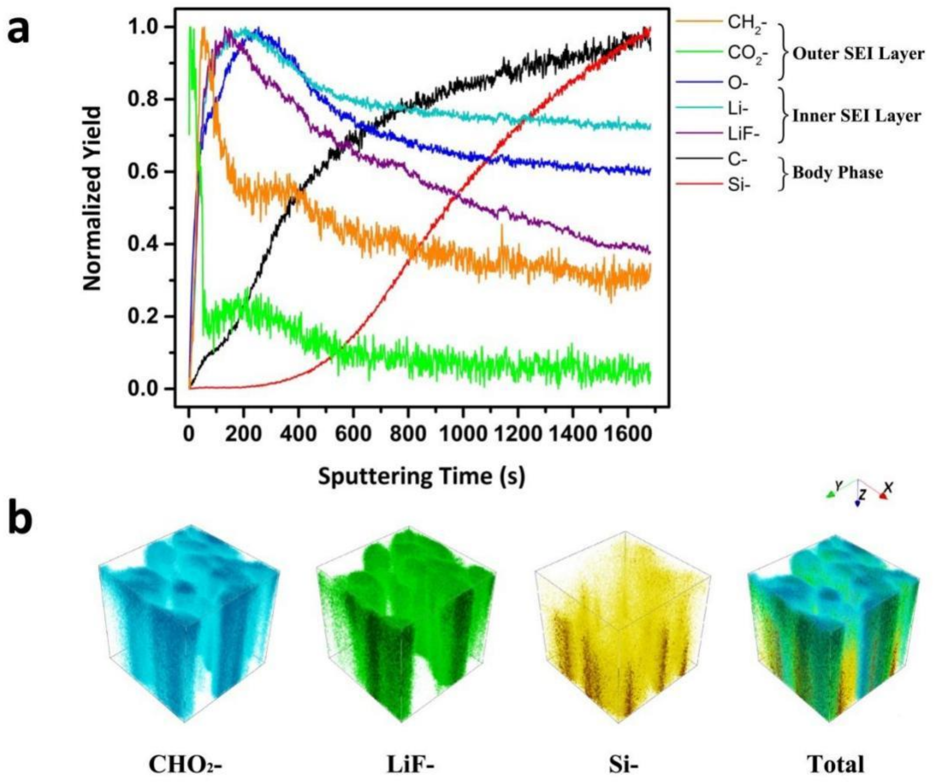

Figure S1. TOF-SIMS analysis of the traditional SEI layer. (a) Normalized (to their maximum) depth profiles of various secondary ions of interest contained in the sputtered volume at the modified layer; (b) Individual spatial distributions of the selected secondary ions (CHO_2-, LiF- and Si-) and 3D, multicolor overlay of representative secondary ions composing the different layers of the interface.

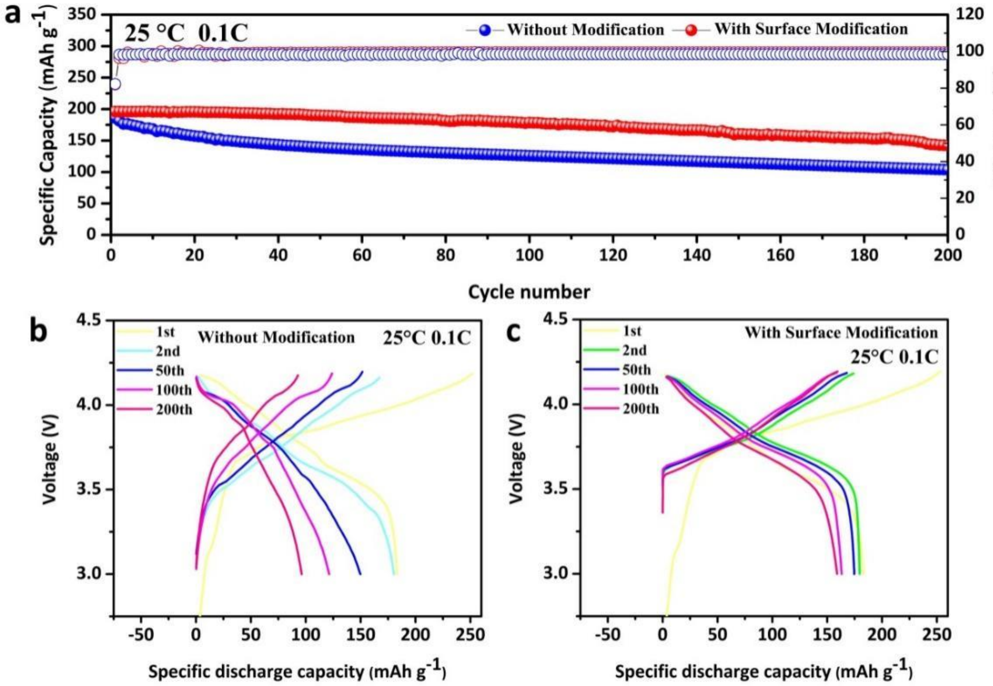

Figure S2. (a) Long cycle performance of two kinds of batteries at room temperature. (b), (c) Charge-discharge curves of two kinds of batteries at room temperature.

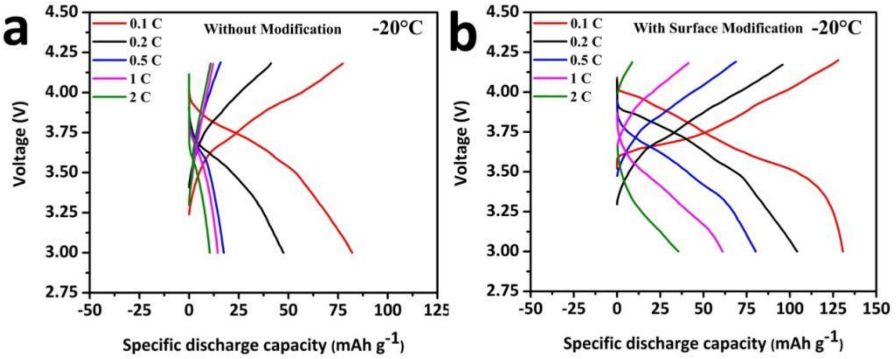

Figure S3. (a), (b) Charge and discharge curves of two kinds of batteries under

different current rates at -20 °C.

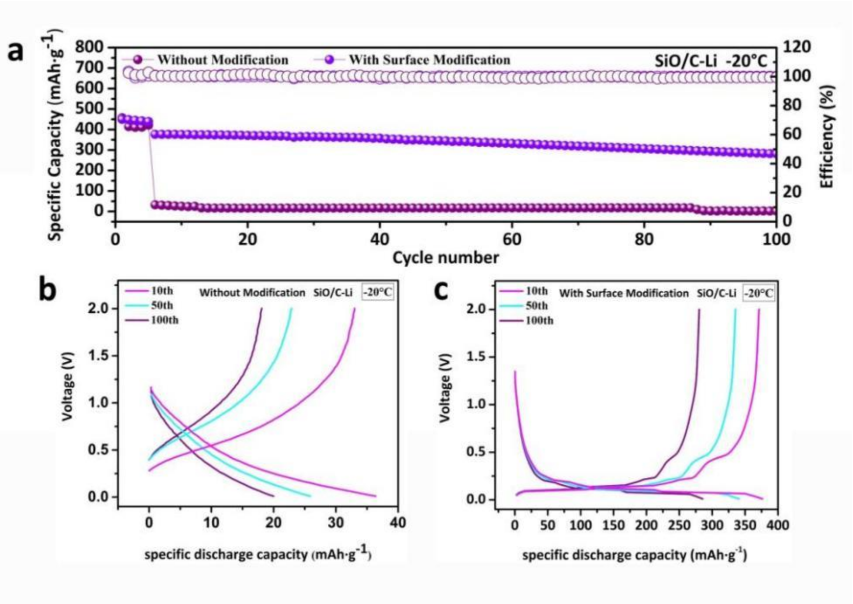

Figure S4. (a) Long cycle performance of SiO/C anode with modified layer and without modified layer at low temperature after being matched with Li respectively. (b), (c) charge-discharge curves of two systems.

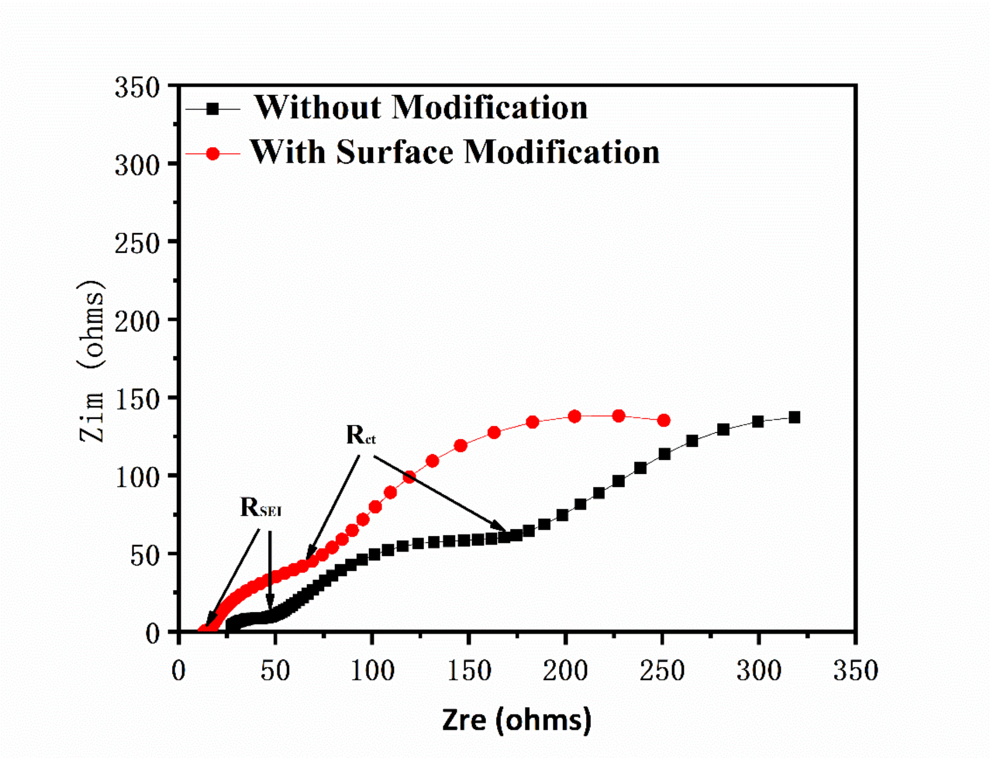

Figure S5. (a) EIS of two types of batteries at -20 °C.

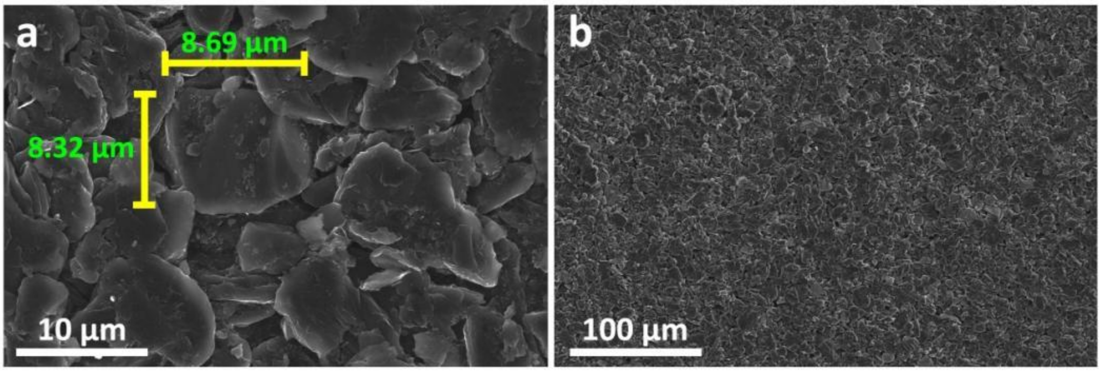

Figure S6. (a), (b) SEM images of the blank SiO/C anode at high magnification and low magnification.

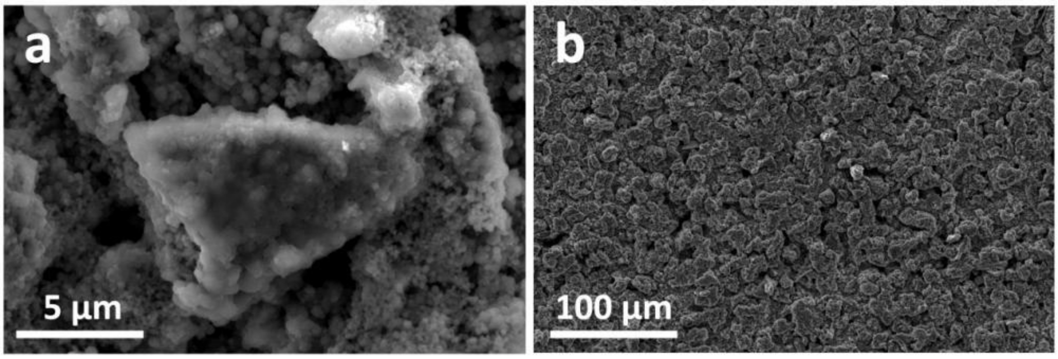

Figure S7. (a), (b) SEM images of the modified SiO/C anode at high magnification and low magnification.

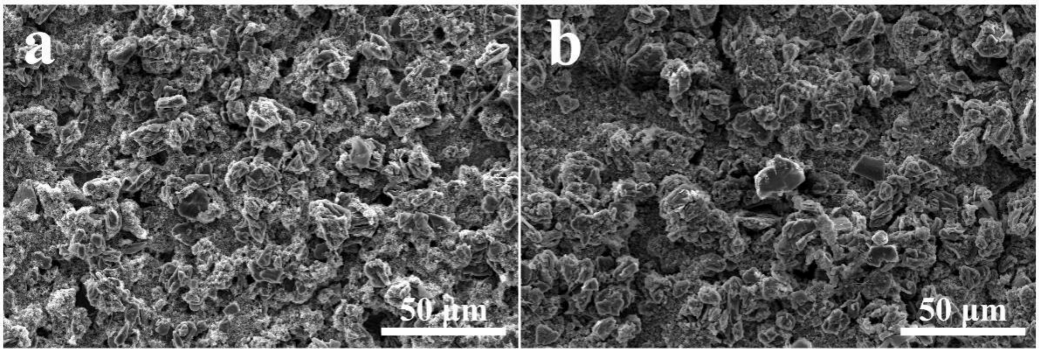

Figure S8. SEM image of SiO/C anode (a) without and (b) with modified layer after 50 cycles at -20 °C.

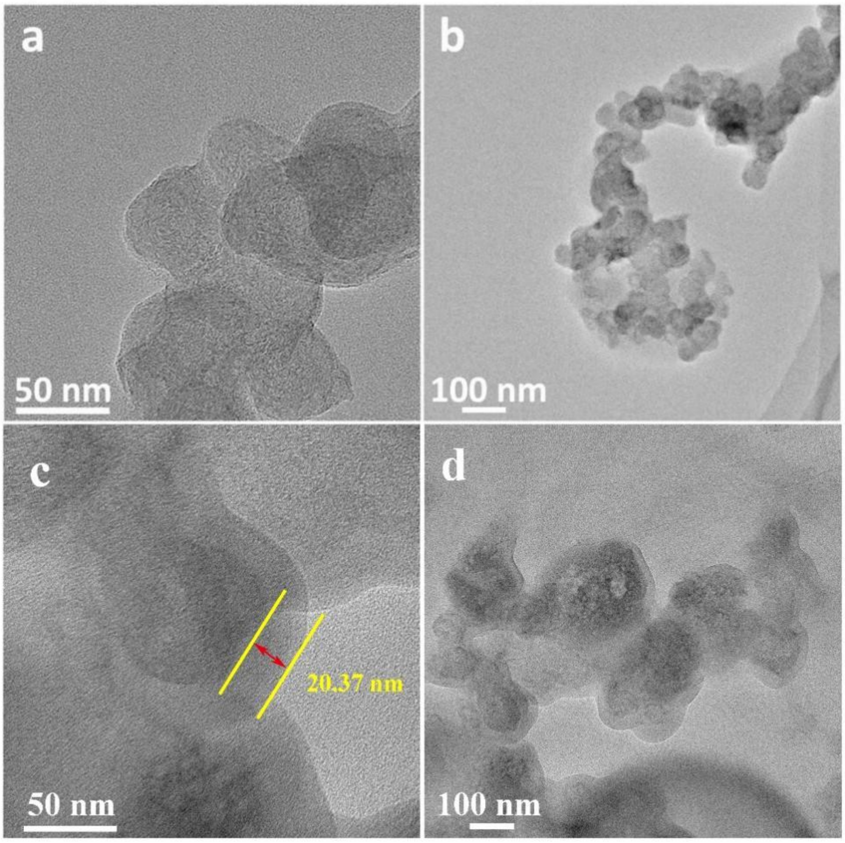

Figure S9. TEM images of (a), (b) the blank and (c), (d) the modified SiO/C anode at high magnification and low magnification.

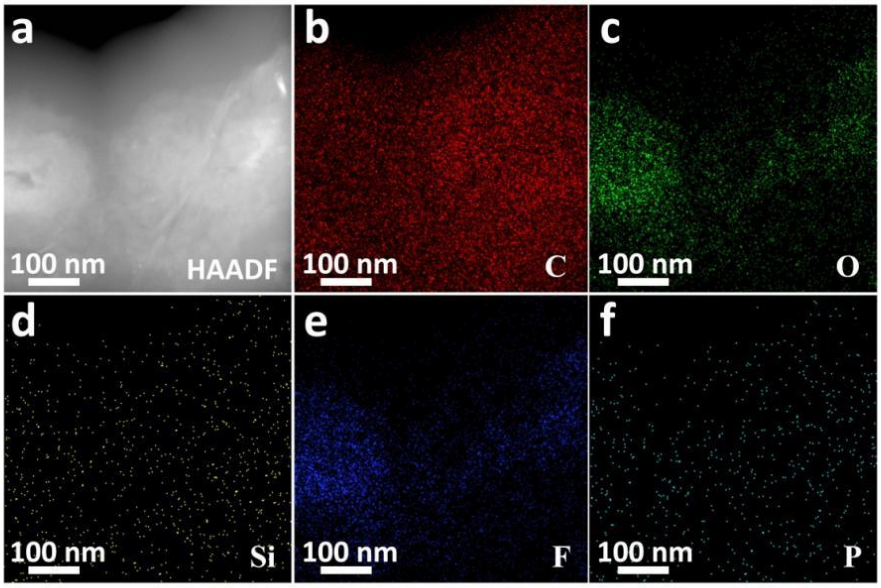

Figure S10. (a) - (f) The EDS images of C, O, Si, F, and P elements corresponding to the SiO/C anode of the brand-new full battery after 50 cycles.

References

[1] M. J. Abraham, T. Murtola, R. Schulz, S. Páll, J. C. Smith, B. Hess, E. Lindahl, *SoftwareX* **2015**, 1-2, 19.

[2] D. Chen, K. P. Taylor, Q. Hall, J. M. Kaplan, *Genetics* **2016**, 204, 1151.

[3] L. S. Dodda, I. Cabeza de Vaca, J. Tirado-Rives, W. L. Jorgensen, *Nucleic Acids Research* **2017**, 45, W331.

[4] T. Lu, F. Chen, *J Comput Chem* **2012**, 33, 580.

[5] L. Martinez, R. Andrade, E. G. Birgin, J. M. Martinez, *J Comput Chem* **2009**, 30, 2157.

[6] G. Bussi, D. Donadio, M. Parrinello, *J Chem Phys* **2007**, 126, 014101.

[7] C. Adamo, V. Barone, *The Journal of Chemical Physics* **1999**, 110, 6158.

[8] F. Weigend, R. Ahlrichs, *Phys Chem Chem Phys* **2005**, 7, 3297.
